# Social Modulation during Songbird Courtship Potentiates Midbrain Dopaminergic Neurons

**DOI:** 10.1371/journal.pone.0003281

**Published:** 2008-10-01

**Authors:** Ya-Chun Huang, Neal A. Hessler

**Affiliations:** Vocal Behavior Mechanisms Laboratory, RIKEN Brain Science Institute, Wako-shi, Saitama, Japan; Chiba University Center for Forensic Mental Health, Japan

## Abstract

Synaptic transmission onto dopaminergic neurons of the mammalian ventral tegmental area (VTA) can be potentiated by acute or chronic exposure to addictive drugs. Because rewarding behavior, such as social affiliation, can activate the same neural circuitry as addictive drugs, we tested whether the intense social interaction of songbird courtship may also potentiate VTA synaptic function. We recorded glutamatergic synaptic currents from VTA of male zebra finches who had experienced distinct social and behavioral conditions during the previous hour. The level of synaptic transmission to VTA neurons, as assayed by the ratio of α-amino-3-hydroxy-5-methyl-4-isoxazolepropionic acid (AMPA) to N-methyl-D-aspartic acid (NMDA) glutamate receptor mediated synaptic currents, was increased after males sang to females, and also after they saw females without singing, but not after they sang while alone. Potentiation after female exposure alone did not appear to result from stress, as it was not blocked by inhibition of glucocorticoid receptors. This potentiation was restricted to synapses of dopaminergic projection neurons, and appeared to be expressed postsynaptically. This study supports a model in which VTA dopaminergic neurons are more strongly activated during singing used for courtship than during non-courtship singing, and thus can provide social context-dependent modulation to forebrain areas. More generally, these results demonstrate that an intense social encounter can trigger the same pathways of neuronal plasticity as addictive drugs.

## Introduction

A wide range of studies in mammals have provided support for a model in which reward is signaled in the brain by increased activity of dopaminergic neurons in VTA, and subsequent phasic dopamine release into forebrain areas [1]–[3]. In mammals, the same circuits can be activated by addictive drugs, which can cause long-lasting changes in function that can disrupt motivated behavior, including social behavior [4]–[7]. One critical alteration after drug use is enhancement of synaptic transmission onto midbrain VTA dopaminergic neurons [8]–[10]. We tested here whether the same synaptic plasticity can also be caused by a natural social situation, courtship singing of male songbirds.

**Figure 1:**
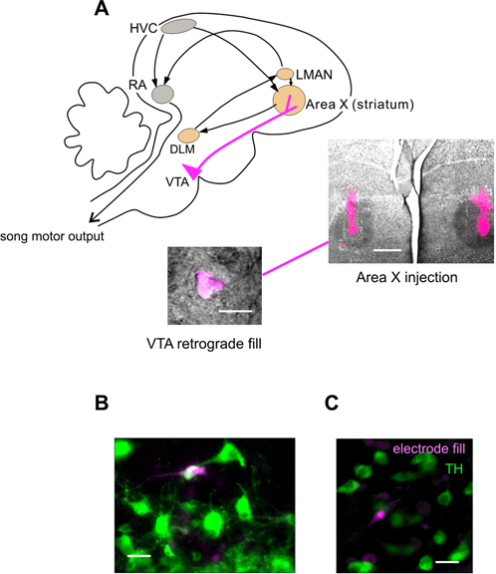
Anatomical identification of VTA cell types. (A) Schematic diagram of sagittal view of singing-related areas in the songbird brain. Motor control nuclei (gray) are critically involved in structuring song output, while nuclei of the anterior forebrain pathway (AFP, orange) are required for song plasticity, and appear to be involved in the communicative function of singing. Area X receives a especially strong dopaminergic input from VTA. Below right is an image of a representative bilateral tracer (Fluoro-ruby) injection in Area X. Image of fluorescent tracer (magenta) is overlaid on image of nissl stain in one brain section including Area X (darkly stained oval nucleus). To the left is an overlay of fluorescently labeled cell body retrogradely labeled by an injection in Area X with IR-DIC image visualized in living brain slice. Scale bars indicate 15 uM (left) and 750 uM (right). (B,C). Post-recording confirmation of cell type by immunohistochemistry and electrode-filling dye. Green label for tyrosine hydroxylase (TH) antibody is overlaid with magenta label of neurons filled with fluorescent dye from the recording electrode (Alexa 568, Molecular Probes) – white signal indicates overlap of two signals. Left panel presents example of post-recording confirmation of TH-positive dopaminergic neuron, and right panel shows example of recording from TH-negative presumptive gamma-aminobutyric acid (GABA) -ergic neuron. Scale bars indicate 30 uM.

A series of previous studies have provided evidence that courtship singing by male songbirds is associated with activation of brain areas likely involved in processing reward signals. In the most frequently studied species, the zebra finch, males produce ‘directed’ song during courtship of a female finch. Males also produce a similar ‘undirected’ song when not in the presence of another bird. These song types can be distinguished by subtle differences – the tempo of a male's directed songs is typically slightly faster than that of his undirected songs, and details of fine acoustic structure are more variable from song to song during undirected singing [11]–[13]. Such differences are important to a singer's critical audience, female zebra finches, who prefer to approach directed songs [14]. The distinct features of undirected songs appear driven by a higher and more variable level of activity in the lateral magnocellular nucleus of the nidopallium (LMAN), which projects to the premotor robust nucleus of the arcopallium (RA; [15]–[17]; Fig. 1A). Inactivation [13] or lesions [18] of LMAN immediately reduce the variability of song output, causing all songs to become more similar to “directed” songs. Recent studies have suggested that modulation of neural activity in LMAN, and the interconnected striatal nucleus Area X, may be in part due to higher levels of dopamine release in Area X during directed singing. When males sing to attract a female, but not when they sing while alone, the level of neural activity and the level of activity-dependent gene expression in a major dopaminergic input, VTA, is selectively modulated [19]–[20], and higher levels of dopamine can be measured in Area X [21] (Fig. 1). In summary, these studies support a model in which singing-related neural activity in the anterior forebrain pathway (Fig. 1; striatal Area X -> dorsal lateral nucleus of the medial thalamus (DLM) -> pallial LMAN) is specifically modulated by dopaminergic input from VTA during directed courtship singing, which reduces the variability of the output of the system to the motor pathway at RA, and biases song output to the higher stereotypy typical of courtship.

However, a critical aspect of this model, that VTA dopaminergic neurons projecting to Area X are more active during directed than undirected singing, has not yet been definitively demonstrated. The physiological study could not confirm the identity of VTA neurons that were active during directed singing, and thus many or all could have been non-dopaminergic neurons [20]. Further, it was not demonstrated that VTA was the source of higher levels of dopamine detected in Area X during directed singing [21].

Here we have taken a new approach to monitor the level of activation of VTA dopaminergic neurons during courtship. In mammals, injection of addictive drugs that increase the firing rate of dopaminergic neurons results in a potentiation of synaptic inputs onto them [8]–[10]. Thus, we hypothesized that if songbird VTA dopaminergic neurons are more active during directed than undirected singing, there may be a similar relative potentiation after directed compared to undirected singing. The relative level of glutamatergic transmission, as assayed by the ratio of AMPA to NMDA receptor mediated excitatory postsynaptic currents (EPSCs), was consistently higher after males had sung during a one hour period to females than after they sang while alone. We further found that a similar potentiation of transmission occurred in males who were exposed to female birds for one hour, but did not sing. As in mammals after drug injections, potentiation was restricted only to dopaminergic projection neurons, and appeared to be expressed as a postsynaptic enhancement of synaptic responses rather than enhanced presynaptic transmitter release. This system should be especially useful for characterizing interactions of rewarding affiliative behaviors with brain reward pathways.

## Results

In all experiments, we recorded synaptic currents of identified neurons in VTA, after male birds had experienced one of four specific social conditions. As in mammals, the VTA of birds contains both dopaminergic projection neurons and non-dopaminergic neurons [22]. These classes are clearly distinguishable by the restriction to dopaminergic neurons of the catecholamine synthetic enzyme, tyrosine hydroxylase (TH; Figure 1B). In order to identify the type of neuron we recorded, dopaminergic and non-dopaminergic neurons were distinguished after recordings by immunohistochemical staining for TH, or VTA projection neurons were identified during the experiment by the presence of a fluorescent tracer injected earlier into a target nucleus, Area X (Figure 1A). As several previous studies found that such retrogradely labeled neurons in VTA had little or no overlap with physiologically [22] or immunohistochemically [20] identified non-dopaminergic neurons, we will refer to them here as presumptively dopaminergic. By these two methods, we monitored synaptic transmission onto 70 identified VTA neurons from 41 birds (52 identified by post-recording immunohistochemical staining, and 18 presumptively dopaminergic projection neurons identified by visual confirmation of retrograde tracer; see Materials and Methods for details).

The strength of glutamatergic synaptic transmission is largely dependent on the number of fast, AMPA-receptor channels at the synapse. A common assay to quantify the strength of individual synapses is the ratio of the AMPA-mediated EPSC to the slow NMDA-mediated EPSC. Although this assay only quantifies the relative synaptic strength at the time slices are prepared, it may be used to make inferences about the past history of a synapse. Potentiation of synaptic transmission is often mediated by a enhanced function of AMPA receptor mediated transmission relative to NMDA receptor mediated transmission [23]–[24]. Thus, transmission at synapses with a high AMPA/NMDA EPSC ratio may have been potentiated recently. In our experiments, we used this assay to quantify the strength of glutamatergic transmission onto VTA neurons after male birds had experienced one of three distinct behavioral conditions in the previous hour. We stimulated afferents rostral to VTA, and thus likely activated inputs from a range of forebrain areas, including the ventral pallidum [25]–[27].

**Figure 2:**
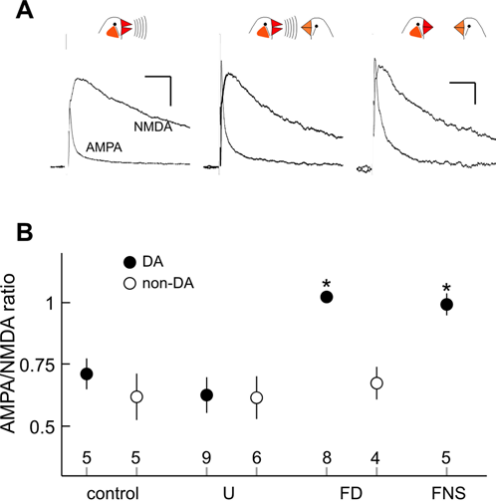
AMPA/NMDA ratio of synapses onto VTA dopaminergic neurons is increased after males are exposed to female birds. (A) Representative plots of average EPSCs mediated by AMPA and NMDA glutamate receptors onto VTA dopaminergic neurons. Left, EPSCs recorded after undirected singing session; Center, after directed singing session; Right, after female exposure without singing. Scale bar in left panel applies to left and middle panels, that in right to the right panel; each indicates 50 ms, 40 pA. (B) Average AMPA/NMDA ratios recorded from dopaminergic (filled) and non-dopaminergic (open) neurons after four behavioral contexts. Mean±s.e.m. are shown for control (colony housed), undirected singing (U), directed singing (FD), and female exposure without singing (FNS) groups (error bar for directed singing FD group is obscured by plot symbol). The number of neurons recorded in each group is indicated above axis.

In Figure 2A, we present representative results of AMPA and NMDA receptor mediated EPSCs recorded from males who had experienced one of three behavioral conditions in the previous hour - singing while alone, singing to a female bird, or being in the presence of a female bird while not singing (see Materials and Methods for details of behavioral conditions). In each plot, the average synaptic current mediated by fast AMPA and slow NMDA glutamate receptors is shown. The AMPA receptor mediated EPSC was revealed by recording after application of the NMDA selective antagonist 2-amino-5-phosphonovalerate (APV), and could be blocked by the application of the AMPA selective antagonist 6-cyano-7-nitroquinoxaline-2,3-dione (CNQX; Figure S1, similar results were seen in 5 recordings from 5 birds).

For these representative experiments, the ratio of the amplitude of AMPA to NMDA receptor mediated EPSCs was higher after a male had sung to a female bird (middle panel, AMPA / NMDA = 1.10) than after a male had sung while alone (left panel, AMPA / NMDA = 0.66; Figure 2A). In order to further characterize the social stimuli necessary for this potentiation, we also examined synaptic function in male birds who were exposed to a female but were actively prevented from singing (one bird did not require external disruption to prevent singing). This situation also caused a relative enhancement of AMPA receptor mediated EPSC (Figure 2A; AMPA / NMDA = 1.02).

Figure 2B presents a summary of results for all experiments. For dopaminergic neurons the AMPA/NMDA ratio was consistently increased after males were exposed to females, and either sang (female directed singing, FD) or didn't sing (female exposure with no singing, FNS), relative to males not exposed to females (control and undirected singing groups; Figure 2B, p<0.001, 1-way analysis of variance (ANOVA), both female groups pairwise higher than control or undirected groups; p<0.01, Holm-Sidak multiple comparisons). In contrast, there was no alteration of synaptic transmission onto non-dopaminergic neurons after any behavioral context (Figure 2B, 1-way ANOVA, p = 0.86, non-dopaminergic neurons were not sampled in the female exposed non-singing group, FNS). Perhaps consistent with the lack of difference in level of potentiation between female-exposed singing and non-singing groups, there was no clear relationship in any singing group between the total amount of singing and the AMPA/NMDA ratio (p = 0.19 to 0.88, linear regression).

**Figure 3:**
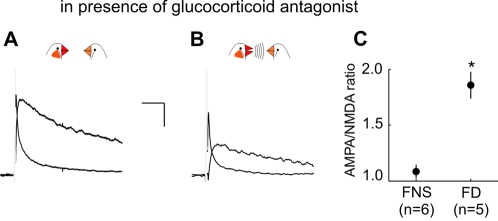
Inhibition of glucocorticoid receptor activation does not block increase of AMPA/NMDA ratio after exposure to female without singing. (A, B) Representative plots of average EPSCs mediated by AMPA and NMDA glutamate receptors onto VTA dopaminergic neurons after not singing (A) and singing (B) in the presence of a female, in males treated with mifepristone. Scale bar indicates 50 msec, 40 pA. (C) Average AMPA/NMDA ratios recorded from males treated with mifepristone after exposure to a female and not singing (FNS, n = 6 recorded from 5 birds) or singing (FD, n = 5 recorded from 5 birds; asterisk indicates p<0.001, t-test).

In mammals, glutamatergic input to dopaminergic neurons can be potentiated after an acutely stressful situation [10]. In our experiments it seemed possible that the potentiation following exposure to a female without singing could have had a similar cause. The first interaction with a nearby female in several weeks may in itself be stressful for some males, such that they were unable to initiate singing. In other birds, the experience of being acutely prevented from singing could cause stress. To test whether such potential stress could have caused a potentiation of transmission, we performed the same experiments after administration of the glucocorticoid antagonist mifepristone, which blocks stress-mediated potentiation in mammals [10]. In males who were exposed to a female without singing, injections of mifepristone did not have a clear effect on the EPSC AMPA/NMDA ratio (Figure 3A, C; FNS with mifepristone: 1.09±0.06, n = 6; FNS without mifepristone: 1.00±0.05 p = 0.24, t-test; this and following data presented as mean±s.e.m.), and thus did not appear to be caused by stress. In contrast, the AMPA/NMDA ratio in males who sang in the presence of a female was clearly increased after males were administered the glucocorticoid antagonist (Figure 3B and 3C; FD with mifepristone: 1.94±0.12, n = 5; FD without mifepristone: 1.02±0.02, p = 0.002, Mann-Whitney rank sum test, n = 5 birds in each experimental group). Although the mechanism by which antagonism of glucocorticoid receptors enhances the AMPA/NMDA ratio after singing is unclear, the presence of this effect only in males who saw females and also sang suggests this behavior is more potent at triggering potentiation than viewing a female only.

**Figure 4:**
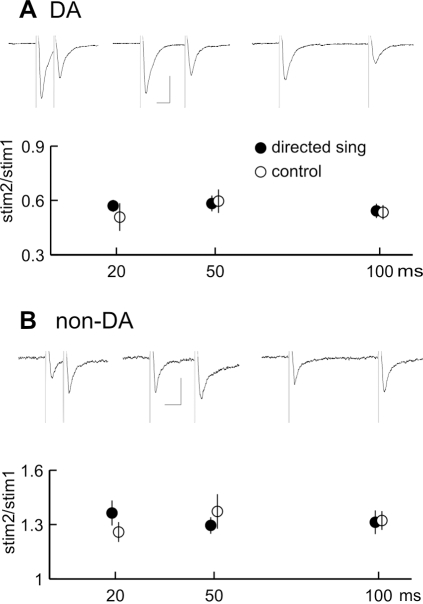
The level of transmitter release is not altered after singing to females. (A, B) Upper section, average EPSC in response to pairs of stimuli given at 20, 50, and 100 ms intervals, respectively. Lower section, average ratio of EPSC amplitude in response to second stimulus relative to first stimulus for similar experiments (DA; n = 13 directed, n = 7 control; non-DA, n = 8 directed, n = 6 control). Scalebar indicates 30 ms, 50 pA, and error bars indicate s.e.m. for each timepoint. Means for directed and control birds are offset slightly on the x-axis for clarity.

In order to test whether the potentiation of the AMPA/NMDA ratio we observed could in part reflect an alteration of transmitter release, we examined the effect of exposure to different social contexts on the magnitude of responses to pairs of electrical stimuli [28]–[29]. As in mammalian VTA [30], synaptic inputs onto dopaminergic and nondopaminergic neurons had distinct response properties. While EPSCs onto dopaminergic neurons are depressed in response to successive stimuli, EPSCs onto non-dopaminergic neurons are enhanced at short stimulation intervals (Fig. 4, n = 5 birds control, 10 birds directed singing). As was previously shown for drug-induced potentiation of mammalian VTA [8], the level of presynaptic facilitation in our experiments was similar after birds had performed directed or undirected singing (Fig. 4; p>0.20, t-test for each comparison). These results suggest that the increased AMPA/NMDA ratio reflected a postsynaptic rather than a presynaptic modification.

We have begun to investigate potential behavioral effects of the potentiation we observed. Assuming a constant level of phasic afferent input during the period of directed singing, the synaptic potentiation could result in increased singing-related activity of VTA dopaminergic neurons from the initial exposure to females to the final period (at the time point we measure synaptic strength). This could result in higher levels of singing-related dopamine release in Area X late during the exposure period compared to early, and thus more strong modulation of activity in Area X and LMAN. Given previous reports that the strength of modulation of activity in these nuclei modulates singing motor output, likely through downstream effects on the motor control nucleus RA [13], [18], we tested whether song structure was acutely altered during the period of exposure to females. If the potentiation we observed can cause altered neural function through several song system brain areas, the last songs males sang could have more “directed-like” structure compared to the initial ones, specifically less variability of fine acoustic features, and a higher singing tempo [11]–[13]. Thus, we compared songs that birds produced within the initial 5 minutes of exposure to females to those produced 40 minutes later.

We were unable to quantify the variability of production of song elements, as has commonly been used previously [13], [18], as accurate estimation of variability required a larger number of undisrupted song repetitions in early and late periods than we could record (simultaneously produced female calls often overlapped with male songs). However, in about half of our birds, we could examine another aspect of singing previously shown to differ between directed and undirected songs, singing tempo. This was quantified by the duration of each successive stereotyped song motif that birds sang (see Material & Methods for details of analysis). For these three birds, there were only small alterations of singing tempo from early to late during female exposure, ranging from a 1.1 % decrease in song motif duration to a 1.8 % increase (for 2 birds, the small differences in duration from early to late were statistically significant, p<0.05 by Mann-Whitney Rank Sum test).

In order to further examine this issue, we performed an additional set of behavioral experiments, in which we compared singing behavior during the early and late female exposure periods, after birds had previously experienced either of two conditions. In one condition (*isolation*), intended to induce a similar social experience as the birds in female-directed and female no singing groups (Fig. 2), males were isolated from other birds (male or female) at least 5 days, and then tested for singing behavior in the presence of females. In the other condition (*social*), males were exposed daily to females for at least 6 days, after which they were tested for singing behavior in the presence of females.

**Figure 5:**
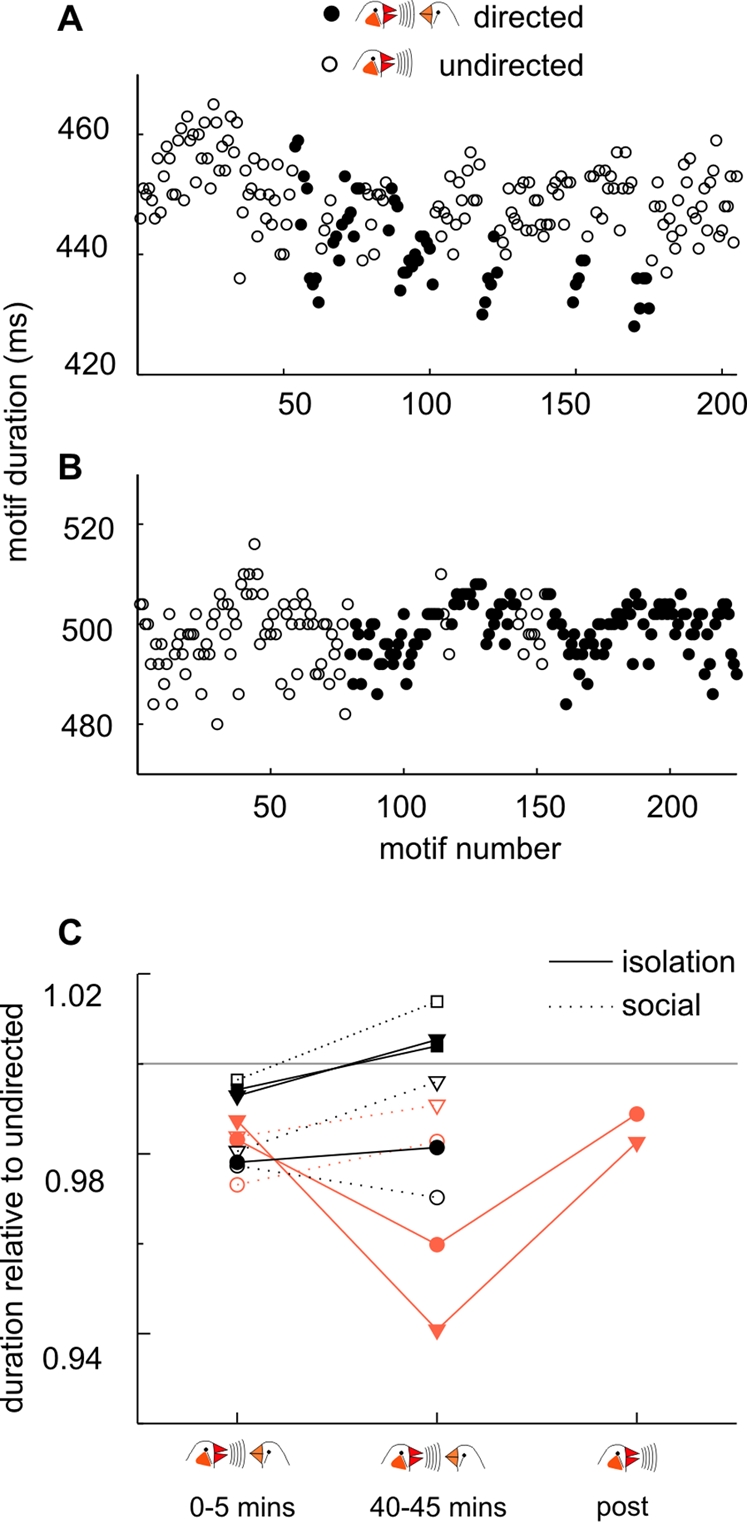
Several days of social isolation can prime short-term song plasticity. (A,B) Duration of successive song motifs produced while alone (empty circles, undirected singing) and targeted to female birds (filled circles, directed). Experiments plotted in A and B represent examples of high (A) and low (B) levels of short-term alteration of song structure during exposure to females. (C). Summary of results from 5 birds recorded while singing to females after 5 days of social isolation (filled symbols, solid lines) or 5 days of daily exposure to females (open symbols, dotted lines). Each point is the plotted the ratio of motif duration relative to undirected song motifs recorded in 10 minutes prior to female presentation, during the three epochs 0–5 minutes after presentation, 40–45 minutes after presentation, and during undirected singing after females were removed. Data from two birds whose directed song motifs were clearly shortened from early to late during female exposure, only after isolation, are displayed in orange. Isolation and social conditions for each bird are plotted with identical symbols and colors.

For some birds, there was a clear decrease of song motif duration during the period of exposure to females, but only in the *isolation* condition. In Figure 5A and B are plotted durations of successive song motifs produced before and during exposure to female birds, for one representative experiment in which motif durations decreased over time (A) and for another experiment in which motif durations were unchanged (B). A summary of results from recordings of 5 birds is shown in panel C. For each recording, the average motif durations in the first and last 5 minutes of exposure to females is plotted divided by the duration of undirected singing motifs just prior to female exposure. While motif durations of 3 birds (plotted in black) had some tendency to increase slightly over the exposure period, motifs of 2 birds (plotted in orange for clarity) were markedly and significantly decreased in duration within 40 minutes of exposure to females. For the experiment shown in panel A, while the duration of song motifs directed to females in the first 5 minutes was not significantly different from previous undirected singing, directed song motifs 40 minutes later were significantly shorter (p<0.05, Mann-Whitney Rank Sum Test, n = 9 motifs 0–5 minutes, n = 10 motifs 40–45 minutes). There was also a significant decrease in motif duration between these periods for one other bird (p<0.05, Mann-Whitney Rank Sum Test, n = 13 0–5 mins, n = 10 40–45 mins).

The degree to which directed motifs were shortened relative to undirected song motifs was striking (0.96 and 0.94), but returned to near pre-exposure level when males sang undirected songs after females were removed (Fig. 5C, post). The accelerated song tempo occurred in both birds within 25 minutes of female exposure (p<0.05, Mann-Whitney Rank Sum Test, mean / pre-exposure = 0.975 and 0.97). Such reductions of motifs duration during female exposure did not occur after the same birds had been exposed to females daily during the previous week (open orange symbols and dotted lines).

## Discussion

Here, we show that an affiliative social behavior can induce a similar synaptic plasticity in VTA dopaminergic neurons as pharmacological overactivation by addictive drugs [8]–[10]. The level of synaptic transmission to VTA dopaminergic neurons was enhanced within an hour after male zebra finches were exposed to female finches, whether they sang to them or not. This potentiation was limited to dopaminergic neurons, and appeared to be expressed by a postsynaptic modification. These results highlight the multifaceted roles of VTA and brain dopaminergic systems, and suggest that such potentiation may be a locus by which social and addictive behaviors interact [31]. Below we discuss these results in relation to previous studies in which a similar plasticity in VTA was caused by addictive drugs, and present possible functions of such plasticity in normal singing and social behavior of male birds.

The enhancement of synaptic function after an intense social experience of a male bird has several features in common with the previously characterized plasticity induced by addictive drugs in mammals. Either an acute or prolonged administration of a variety of addictive drugs such as amphetamine and cocaine can also potentiate glutamatergic transmission in VTA, restricted to dopaminergic neurons [8]–[10]. Further, the relative degree of potentiation we observed here is in the range of that previous reported caused by injection of addictive drugs [8]–[10]. While most previous studies monitored potentiation one day after drug injection [8], [10], [32], one showed that potentiation was initiated only two hours after drug injection [9]. Here we found that input to VTA dopaminergic neurons could be potentiated after only one hour of exposure to a female, with or without singing. We found that, as in mammals, potentiation in VTA was restricted to dopaminergic neurons. We quantified the level of synaptic potentiation here by measuring the increase in the relative size of AMPA to NMDA glutamate receptor synaptic currents. While this likely reflected an enhancement of synaptic transmission by an increase of postsynaptic AMPA receptors, it remains possible that a relative reduction of NMDA mediated currents may also contribute to these results. Further studies, for example quantifying the amount of receptors by immunocytochemistry, will be required to resolve this issue. Although singing to females appeared to cause excitation of inhibitory interneurons in zebra finch males [19]–[20], we could find no potentiation of synapses onto non-dopaminergic neurons, similar to findings in mammals [8]. Thus, it seems likely that dopaminergic neurons in VTA possess specific molecular pathways of plasticity lacking in other VTA neurons.

These results are especially interesting in relation to recent studies of courtship in the same species of songbird, suggesting that this behavior activates brain systems involved in reward processing. In brief, a range of types of experiments have supported a model in which singing-related neuronal activity in several forebrain nuclei, including the striatal nucleus Area X, is selectively modulated when males sing to females and not when they sing while alone, by selective input of dopamine from VTA during the highly motivated courtship behavior. Initial studies found that the level and pattern of neural activity [16] and expression of the immediate early gene egr-1 (also known as zif268, ZENK, and NGF [15]) are strongly dependent on the social context in which males sing in the anterior forebrain nuclei LMAN and Area X, as well as in the motor nucleus RA. Further studies found that the level of expression of several immediate early genes in VTA, as well as other motivation related areas, can be modulated by the specific social context in which males sing [20], [33]. More direct evidence that forebrain modulation may arise from VTA input was provided by recent reports that VTA neuronal activity is selectively modulated during directed but not undirected singing [19]. The results presented here, that VTA dopaminergic neurons projecting to Area X are strongly activated during directed singing, provide the strongest direct evidence that forebrain modulation during courtship singing can be mediated by a dopamine signal from VTA.

In contrast to this conclusion, previous anatomical [20] and physiological [19] studies provided some evidence that VTA neurons active during courtship were mainly non-dopaminergic, and no clear evidence was presented that dopaminergic projection neurons are similarly modulated. However, it remained possible that while non-dopaminergic neurons are activated during courtship singing, dopaminergic projection neurons in the same nucleus may be as well, but they may not have been sampled in physiological recordings [19], or may not possess the molecular pathway leading to expression specific immediate early genes [20]. While this and many previous studies suggest that modulation of forebrain song system activity and singing output modulation by social context is dependent on activation of dopamine systems, other neuromodulators such as norepinephrine may also be involved [34]–[35].

The role of such potentiation of dopaminergic function in the normal life of a male bird remains to be elucidated. We have considered several possible functions. First, the potentiation of glutamatergic input to VTA dopamine neurons we observed here could occur in the natural environment, and suggests that such social interaction with female birds may be very rewarding. In wild zebra finches, such frequent directed singing should be mainly restricted to the period prior to mating, which occurs at intervals of about several months [36]. In intervals between such mating periods, males mainly produce the non-courtship related undirected songs [36]–[38] – the type that did not cause potentiation of dopaminergic neurons in this study. Thus, dopaminergic function could be selectively enhanced during mating periods, and may decrease during non-mating intervals. The potentiation after singing to a female may serve to reinforce courtship singing behavior that is important for affiliation in this mainly monogamous species, and may be related to the previous characterized requirement of dopaminergic enhancement in initiation of pair bonding in mammals, as exemplified by studies of prairie voles [39]–[40].

An additional possibility is that the potentiation we observed was above the range that occurs in the normal life of a bird, because the social experience may have been unnaturally intense. This possibility is suggested by the similar level of potentiation caused by singing to a female, and by only seeing a female while not singing. In our experiments, male birds had been isolated from contact with any female for several weeks - a condition not likely to occur in the normal life of a male zebra finch [36]. After such a prolonged isolation, the experience of singing to a female, or even just being in the presence of a female, may activate brain reward systems beyond the extent that occurs in nature. Related to this issue, a previous study found that activity of single units in VTA was modulated to some extent by the presence of a female alone, but was consistently more strongly modulated when a male also sang to the female [19]. One possibly important distinction between the acute recording study and this one is that in the previous study males had been recently exposed to a female within several minutes, while in this study males were exposed to females for the first time in several weeks. Thus, it is possible that social interaction with a female bird activates the dopaminergic system in a graded manner, normally at a higher level when males sing to a female than when they only see a female, but in abnormally isolated males the sight of a female alone may trigger the same activation. An additional result supporting a higher level of stimulation provided by the context in which males both saw a female and sang to her is given by the result of experiment in which males performed both behaviors after block of glucocorticoid receptors. The level of potentiation in birds who saw a female and didn't sing was similar both in the absence and presence of glucocorticoid antagonism. However, when males sang to a female during glucocorticoid block, the level of potentiation was further enhanced beyond that when seeing a female alone.

Although we could not detect an acute behavioral effect of such synaptic potentiation in birds in which it was measured, we did observe some birds in behavioral experiments whose song clearly became faster during exposure to females. While further study is required, such approaches may allow an inference of acute brain plasticity based on short-term behavioral modulation. A further advantage of such behavioral experiments is that, as we did here, it is possible to test individual birds a number of times, and examine possible effects of previous social conditions on behavioral plasticity. The clear distinction in several birds between acute song shortening after several days of isolation and no shortening after having more social experience may have several important implications. First, it suggests that the baseline tone of the dopaminergic system, or more general motivational systems, may be readily modulated in individuals based on their previous experience, as has been widely studied in mammals related to social isolation and social defeat, for example [41]–[43]. These results also suggest that studies of such highly motivated behaviors as courtship should take care to control the social interaction history of experimental subjects. It should be interesting in further studies to also examine the target of directed singing – the female bird. As a previous study showed that females prefer to approach directed songs rather than undirected ones [14], their motivation may increase further as males' songs become more directed-like. Since hearing the mate's song appears rewarding to female birds, it may be that their brain reward system, and dopaminergic function, may be activated in parallel with the singing male during courtship, in a positive feedback reward loop.

It is interesting to speculate that in mammals, as well, especially rewarding natural events may also cause a similar synaptic potentiation of VTA function. In humans and other mammals, sexual behavior, the sight of a partner, and chocolate can increase activation of VTA [44]–[47], and sexual interactions cause dopamine release in striatal areas [48]–[49]. These results presented here further suggest that widely distinct groups of animals may experience similar emotional states while undergoing analogous types of social interaction.

## Materials and Methods

### Subjects

Forty-one adult male zebra finches (>90 days old), either born in our breeding colony, donated by H. Sakaguchi (Dokkyo University), or purchased locally, were used. All experimental procedures were in accord with RIKEN BSI guidelines and were approved by the RIKEN Animal Experiments Committee.

### Behavioral groups and analysis

Prior to experiments, all birds were housed for at least two months in communal cages containing other males, in a room with about one hundred caged birds. Females were visible at a distance of about 1 meter, and thus should not have been a target of directed singing by experimental male birds. Birds were assigned to one of four behavioral conditions. Control birds were removed from the communal room and immediately sacrificed for slice preparation. The immediately prior singing behavior of these individuals was not monitored. Groups U: undirected singing, FD: female directed; and FNS: exposure to female without singing, were removed from the communal room and housed in small cages (22×28×32 cm) in a sound attenuation chamber (60×50×50 cm), alone or in the presence of other caged males. Two to three weeks later, birds were first moved to an individual housing cage for at least one day in isolation (range 1–5, mean 3.7 days), and the next day moved to the experimental chamber the day before the recording session began. For the U group, birds remained alone, and singing behavior was recorded for a period of 45 minutes. For the FD group, a cage containing 2 female birds was placed next to the male in the recording chamber, in order to induce the male to produce directed courtship song. Females were intermittently visible and occluded by a curtain in order to minimize habituation and maintain high levels of singing. Males in this group were in visual contact with females for approximately 15 - 25 minutes. Birds in mifepristone FD and FNS groups were injected subcutaneously with 50 µl drug (10 mg/ml in dH_2_O, Sigma) 15 minutes before the behavioral experiment was performed as above. This dosage is similar to that previously used in mammalian [10] and avian studies [50] to block stress responses. Birds in FD groups were monitored on video to ensure that their singing was targeted to the female bird. Birds in FNS group were placed next to a female as above, and either did not sing spontaneously or were actively prevented from singing by disrupting them before they began to sing. Singing was recorded online and analyzed using Avisoft-Recorder (Berlin, Germany). Zebra finch song consists of a variable number of repeated song “motifs” – stereotyped sequences of discrete vocal elements lasting up to about one second [36]. In order to quantify singing level, we counted the number of song motifs each bird produced in the recording session. For all singing groups, only birds who sang more than 45 motifs in the 45 minute session were used. (FD birds sang an average of 155.9±55, U birds = 163.4±18, and FD+mifepristone = 173±73 motifs).

Additional birds for which only behavioral analysis was performed were treated in two conditions before singing measurement. In the isolation condition, males were placed in an individual cage in a sound isolation chamber with no other birds. After 5–6 days, birds were transferred to the sound recording chamber. In the social condition, males were placed in an individual cage in a sound chamber with no other birds, and a cage containing females was placed inside the chamber for about one hour daily for 6 days, after which the male was transferred to the sound recording chamber. The next day, after at least 15 minutes of undirected singing was recorded, females were presented to the male for 50 min to elicit directed singing. During this period, a cage containing two female finches was placed inside the chamber next to the male and removed from the chamber at about 5 minute intervals. During female presentation, directed singing behavior was confirmed by video observation. When females were not present, males occasionally sang undirected song. In order to quantify singing tempo, the duration of motifs was measured by a sound amplitude level threshold-crossing routine. Motifs whose beginning or end were obscured by female vocalizations were not used. Statistical comparisons between durations of motifs in different singing periods were typically made with a Mann-Whitney Rank Sum test (SigmaStat).

### Slice preparation

Birds were deeply anesthesetized by isoflurane inhalation and decapitated. The brain was removed and then mounted in a 2.5% agarose gel (Low Gelling Temperature; Wako Pure Chemical Industries, Ltd., Japan) prepared with Tyrode's solution (in mM: 134 NaCl, 3 KCl, 10 HEPES, 4 NaOH, 2 CaCl_2_, 1 MgCl_2_). Slices were made in ice-cold artificial cerebrospinal fluid (ACSF) containing (in mM) 85 NaCl, 75 Sucrose, 2.5 KCl, 1.25 NaH_2_PO4, 4 MgSO_4_, 0.5 CaCl_2_, 25 NaHCO_3_, and 25 Glucose. Coronal slices (250 µm) were cut with a vibrating slicer (Leica VT1000S, Nussloch, Germany), incubated in an interface chamber for at least 1 hour in the slicing solution at room temperature (22–24°C), and then transferred into the recording chamber. During recording, ACSF contained (in mM) 119 NaCl, 2.5 KCl, 1 NaH_2_PO_4_, 1.3 MgSO_4_. 2.5 CaCl_2_, 26.2 NaHCO_3_, 11 Glucose, bubbled with 95% O2 / 5% CO2.

### Electrophysiology

Whole cell recordings of neurons in VTA were made under visual control by infrared-differential contrast video microscopy (Olympus BX-51WI, Tokyo, Japan). All recordings were performed at room temperature with an Axopatch 200B amplifier (Axon Instruments, Foster City, CA, USA), from about 2–7 hours after slice preparation. For recording AMPA and NMDA mediated EPSCs, the pipette was filled with a solution containing (in mM) 120 CsCH_3_SO_3_, 20 HEPES, 0.4 EGTA, 2.8 NaCl, 5 TEA-Cl, 2 MgCl_2_, 2.5 MgATP and 0.3 GTP, pH∼7.2 (with CsOH) and osmolarity 290–300 mOsm. Synaptic responses were elicited by a concentric bipolar electrodes (impedance ∼200 kOhm, FHC, Brunswick, ME, USA) controlled by a MASTER-8 stimulator (2–4 mA , A.M.P.I., Jerusalem, Israel) which was positioned approximately 150 µm rostral to the recording electrode. (-)-Bicuculline methobromide (BMI, 10 µM, Tocris, Bristol, UK) was present in the perfusion solution to eliminate GABA_A_ receptor-mediated IPSCs. The amplitude of AMPA to NMDA receptor mediated EPSCs were obtained from neurons voltage-clamped at +40mV. After recording a stable EPSC for 5 minutes, the AMPA-receptor mediated EPSC was isolated by bath application of 50 µM D-APV for 5–10 minutes. Digital subtraction of AMPA-receptor EPSC from the total EPSC represented NMDA-receptor EPSC. Data were quantified by measuring the peak amplitude of average of 15–20 EPSCs for each type of EPSC response. For paired-pulse experiments, pairs of stimuli were given at intervals of 20, 50, and 100 msec, with intertrial intervals of 10 sec, and recordings were made at a holding potential of −70 mV. Averages of 10 trials were made for quantification. Data acquisition and analysis were performed using a digitizer (DigiData 1322A, Axon Instruments) and the analysis software pClamp 9 (Axon Instruments) Origin Pro (OriginLab Co.), and MATLAB (Mathworks). Statistical comparisons were made with SigmaStat (Systat Software).

### Anatomical analysis

For post-recording identification of neuron type, cells were filled by including the fluorescent dye Alexa 568 in the recording pipette (0.1 mM, carboxylic acid, succinimidyl ester mixed isomers: fluoro-ruby, Molecular Probes). After recording, each slice was fixed by immersion in 4 % paraformaldehyde in 0.1 M phosphate buffer (PB) for 12–24 hr at 4°C. For TH immunostaining, slices were incubated with 10% normal goat serum and 0.5% Triton-X in 0.1M PBS for 30 minutes, and subsequently with a mouse anti-TH monoclonal antibody (1:1000; Chemicon, Temecula, CA), 1% goat serum, and 0.5% Triton-X in 0.1M PBS overnight at 4°C. After that, slices were washed by 0.1M PBS three times for 10 minutes, then incubated with Alexa 488 (1:1000; Molecular Probes) goat anti-mouse secondary antibody at room temperature for 2 hours and followed by another set of washes. Images were obtained by Olympus BX60 microscope and contrast was adjusted with Slidebook 4.1 imaging software.

For identification of presumptive dopaminergic neurons [20], [22] during the recording session, retrograde tracer was injected into Area X before experiments. Birds were positioned in a stereotaxic frame under isoflurane anesthesia, and 1 µl of tracer (10% dextran, tetramethylrhodamine dissolved in deionized water; Molecular Probes) was injected bilaterally targeted at Area X: 5.1 mm anterior and 1.7 mm lateral to the divergence of the central sinus at the border of the forebrain and cerebellum, 3 mm below the brain surface. After recovery, birds were returned to their cage for 6 to 9 days before a behavioral experiment. During slice preparation, slices were quickly moved to a dark holding chamber where they were kept to reduce bleaching of signal. Fluorescent signal was detected with a cooled CCD camera (Hamamatsu ORCA-ER, Hamamatsu Photonics K.K., Japan) and AquaCosmos 2.6 software. Patching onto and recording from filled projection neurons was visually guided by alternating the camera's signal between fluorescent and infrared-differential interference contrast (IR-DIC) modes. Accurate tracer injection into Area X was confirmed by examining anterior brain sections containing Area X (40 uM; 10/12 birds; e.g., see Figure 1B).

## Supporting Information

Figure S1 Averages of 30 successive EPSCs for a representative experiment, recorded at a holding potential of +40 mV, in the presence of 10 uM BMI, BMI+50 uM APV, and BMI+APV+10 uM CNQX.
(7.90 MB TIF)
