# Supplementary figures and images for: Social Modulation during Songbird Courtship Potentiates Midbrain Dopaminergic Neurons
Source: PLoS One. 2008 Oct 1;3(10):e3281. doi: 10.1371/journal.pone.0003281 (PMC2533700; doi:10.1371/journal.pone.0003281)

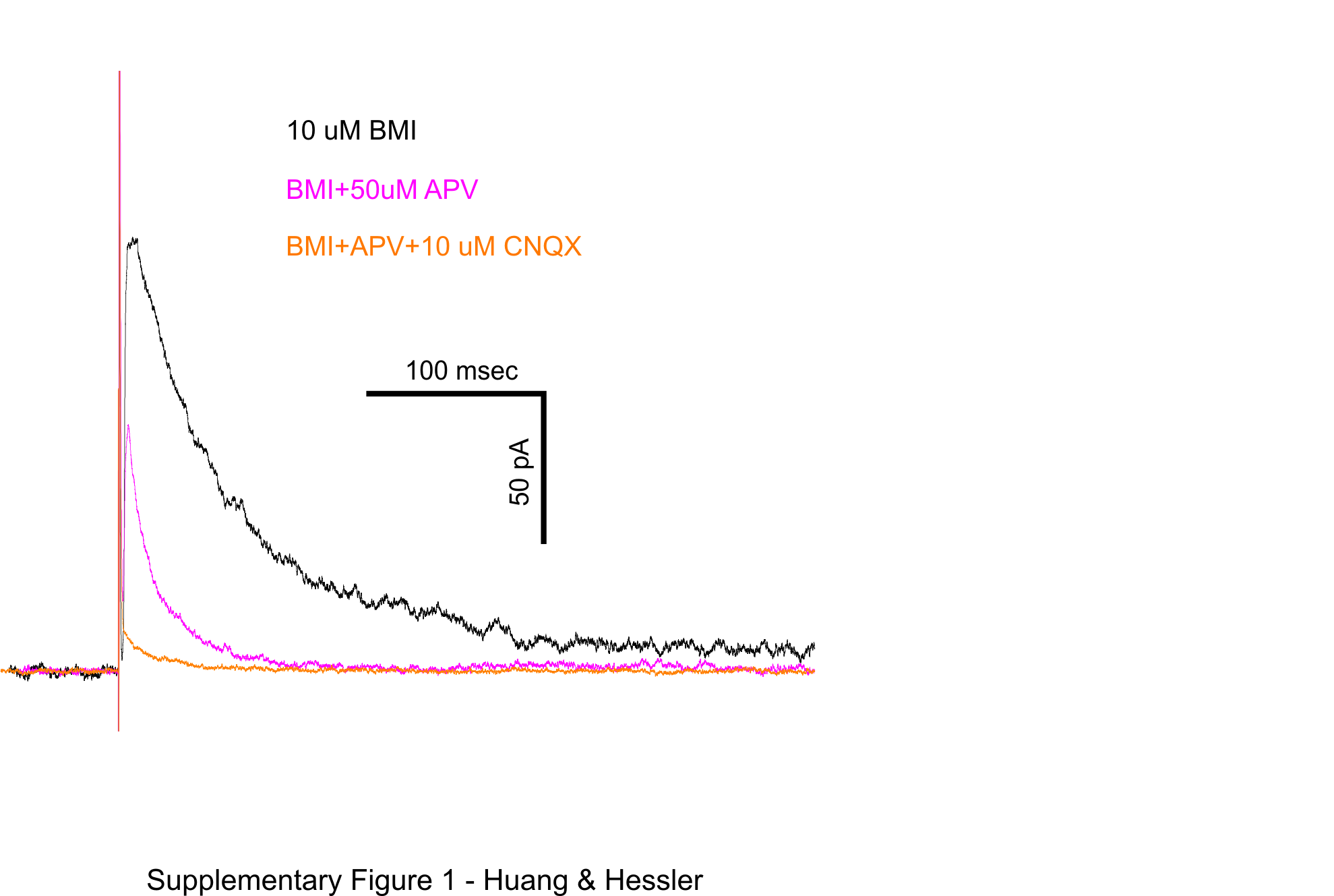

Supplement: Figure S1 — Averages of 30 successive EPSCs for a representative experiment, recorded at a holding potential of +40 mV, in the presence of 10 uM BMI, BMI+50 uM APV, and BMI+APV+10 uM CNQX. (7.90 MB TIF) [file pone.0003281.s001.tif]
